# Multispectral Portable Fibre-Optic Reflectometer for the Classification of the Origin of Chicken Eggshells in the Case of *Mycoplasma synoviae* Infections

**DOI:** 10.3390/s22228690

**Published:** 2022-11-10

**Authors:** Anna Pakuła, Wojciech Żołnowski, Sławomir Paśko, Olimpia Kursa, Paweł Marć, Leszek R. Jaroszewicz

**Affiliations:** 1 Institute of Micromechanics and Photonics, Warsaw University of Technology, Św. A. Boboli 8, 02-525 Warsaw, Poland; 2 Faculty of New Technologies and Chemistry, Military University of Technology, ul. gen. Sylwestra Kaliskiego 2, 00-908 Warsaw, Poland; 3 Institute of Optoelectronics, Military University of Technology, ul. gen. Sylwestra Kaliskiego 2, 00-908 Warsaw, Poland; 4 Department of Poultry Diseases, National Veterinary Research Institute, Al. Partyzantów 57, 24-100 Puławy, Poland

**Keywords:** optical reflectometry, machine learning, fibre-optic sensors, *Mycoplasma synoviae*, origin classification, support vector machine algorithm

## Abstract

The proper classification of the origins of food products is a crucial issue all over the world nowadays. In this paper, the authors present a device—a multispectral portable fibre-optic reflectometer and signal processing patch—together with a machine-learning algorithm for the classification of the origins of chicken eggshells in the case of *Mycoplasma synoviae* infection. The sensor device was developed based on previous studies with a continuous spectrum in transmittance and selected spectral lines in reflectance. In the described case, the sensor is based on the integration of reflected spectral data from short spectral bands from the VIS and NIR region, which are produced by single-colour LEDs and introduced to the sample via a fibre bundle. The measurement is carried out in a sequence, and the reflected signal is pre-processed to be put in the machine learning algorithm. The support vector machine algorithm is used together with three different types of data normalization. The obtained results of the F-score factor for classification of the origins of samples show that the percentages of eggs coming from *Mycoplasma synoviae* infected hens are up to 87% for white and 96% for brown eggshells.

## 1. Introduction

Detecting chemical substances, microbiologic parasites and the contaminations of pathogens is crucial to preventing the spreading of diseases. Mass-produced food is constantly exposed to these problems. Therefore, developing new measurement methods allowing for an early detection of possible infections is necessary. This article proposes a new testing method that can be applied to flocks of livestock such as poultry, chickens in particular. An example of a worldwide spread pathogen that causes substantial economic losses in the poultry industry is *Mycoplasma synoviae* (MS) [1,2,3,4].

MS is one of the bacteria that can be transmitted vertically to eggs. Its presence in the oviducts of chickens causes the appearance of a changed eggshell surface, thinning and increased transparency in different parts of eggshells [3,4]. Since the identification of this bacteria, various detection methods have been developed. Several serological methods are currently used to diagnose MS infection, i.e., the serum plate agglutination test (SPA), enzyme-linked immunosorbent assays (ELISA) and the hemagglutination inhibition test (HI) [5,6,7,8,9]. For the identification of MS, culture methods that require a pleuropneumonia-like organisms (PPLO) broth can also be used; however, they are very time-consuming (up to 28 days) [1]. Molecular methods are the most commonly used to detect MS infections: polymerase chain reaction (PCR) [10,11] and its modifications, real-time PCR, multiplex PCR, loop-mediated isothermal amplification (LAMP) [8,11,12,13,14,15] and polymerase spiral reaction (PSR) [2]. Furthermore, for example, PSR is 100 times more sensitive than PCR, and is characterised by a higher positive rate—69.9%—than ELISA—65.3%.

Another approach is indirect optical spectroscopy, in which the biological objects are classified by their origin, i.e., different honey types [16] or eggs from MS-infected chickens or healthy chickens [17,18]. Due to the large variety of biological samples and their variations within one class, there are many different approaches for spectral data analysis. Principal component analysis (PCA) is one of the methods used [19,20]. Unfortunately, due to such factors as subraces, hens ages, egg colouring, diet and climate, the variety of eggshells is so vast that standard PCA algorithms are ineffective. Other approaches used as classifiers, such as the spanning tree together with various methods of data reduction, may be used [18]. Using this classifier in the discussed case results in multiple level tree analyses. In this study, the authors found the machine learning algorithms to be the most effective.

Two configurations of optical systems with transmitted or reflected light were used. For the transmitted light analysis on chicken eggs, the authors obtained an accuracy of 88.8% for white eggshells [17]. Due to reflective light used, measurements did not require the destruction of the egg, and are therefore more likely to be applied in the industry. Different reflective properties characterise eggshells originating from infected and non-infected chickens. As proven in [21], detecting changes caused by MS infection in a chicken flock is possible by analysing the back-reflected signals from an eggshell of selected spectral wavelengths of a white-light source. The use of machine-learning algorithms allowed for the distinguishing of the tested samples of different origins with reasonable probability. The F-scores in these cases were 95.75% for white eggshells and 86.21% for brown eggshells [21].

An improvement to the reflective methods presented in this paper is to use selected single-colour LEDs instead of a broadband light source and an application fibre bundle, making this device a portable multispectral fibre-optics reflectometer.

For more complex cases, for instance when information about molecules is required, deep-learning methods are sometimes used. Such an approach can be seen in the work of Gosh et al., wherein they applied the method to predict molecular excitation spectra [22]. The results they obtained showed that this kind of network can learn spectra with up to 97% accuracy and can infer the spectra from the molecular spectra only. A similar application of deep learning is presented by Joung et al [15]. Using their method, they predicted seven related-to-optics properties of organic compounds. Drugs can also be identified with this method. This was proven by Ting et al. [23]. This approach allows, as it can be seen, for efficient work in this area. To analyse the distribution of any material spectra, less complex machine-learning methods are mostly used such as those shown above.

## 2. Portable Multispectral Fibre-Optic Reflectometer

The general concept of the measurement is based on the sequential illumination of a tested object with light from the specific VIS regions and the simultaneous detection of the back-reflected light by a photodetector unit. As proven by [21], detecting changes caused by MS infection in a chicken flock is possible by analysing signals back-reflected from an eggshell of the selected spectral wavelengths of a white light source. Different reflective properties characterise eggshells originating from infected or non-infected chickens. The use of machine learning algorithms allows for the distinguishing of the tested samples of different origins with reasonable probability. The described experiment shows that selected single-colour LEDs can replace the broadband light source with non-overlapping spectral characteristics.

**Figure 1:**
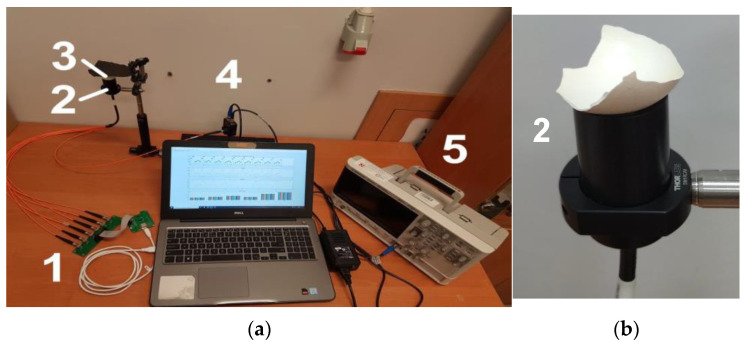
The images of: (**a**) the laboratory portable multispectral fibre-optic reflectometer setup: an electronic driving unit with a set of six LEDs integrated into an electronic board and a 1 × 7 fun-out fibre-optic bundle (1), measuring head (2), tested object with a shade (3), a photodetector unit (4) and an oscilloscope (5); (**b**) measuring head with the tested piece of the eggshell.

The multispectral optical fibre reflectometer (Figure 1a) presented in this paper was a portable device due to being a fibre-optics bundle appliance. The system is based on a reflected light analysis, as in ref. [21]. Still, instead of light propagation in free space and using a continuous broadband white-light source, it used six single-colour LEDs and a fibre bundle that enabled light propagation without external disturbances. The image of the laboratory version of the system is shown in Figure 1. It consisted of an electronic driving unit with a set of six LEDs integrated into an electronic board and linked to a 1 × 7 fun-out fibre-optic bundle (1), a measuring head (2), a tested object, a shade (3) and a photodetector unit (4); the signal was processed by an oscilloscope (5) and driven by a central unit—here, the PC.

The optical part of the setup was built as follows: six single-colour LEDs were connected to the six inputs of the fibre bundle, and the remaining one (located in the middle of the bundle) was used as a receiver of the light back-reflected from the tested object. The measuring head (Figure 1b), on which the sample/tested object was placed through gravity’s force, was equipped with an optic-fibre collimator and optomechanical elements.

The electronic driving unit had a modular design: a control module and a LED sources module. Such a solution was dictated by the desire to allow for the further development and modification of the electronic system without interfering with the optical system. Both modules were made using the multilayer PCB technology.

The control module was the microcontroller used to build the device was a Seeduino XIAO from the Seedstudio company. The power supply and the communication of the microcontroller were implemented via a USB-C connector. Using CircuitPython from Adafruit, the algorithm that controls the module was written in Python.

The LED module provided stable mounting and a power supply for each diode due to the fixtures attached directly to the board, and the diodes were inseparably connected.

**Figure 2:**
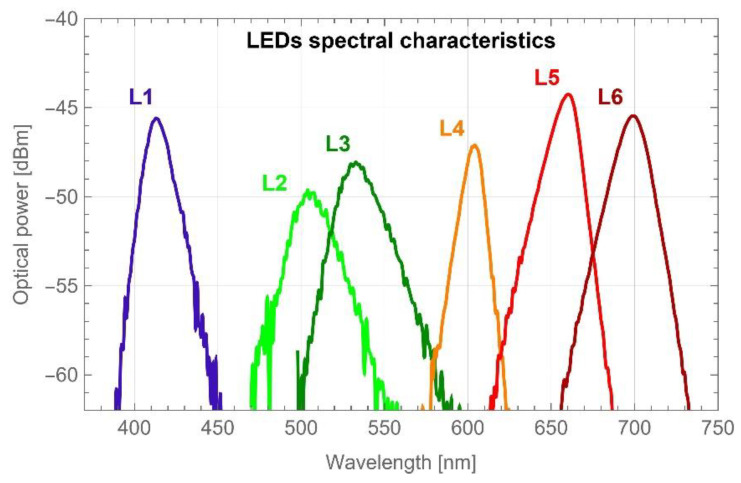
Measured spectral characteristics of all LEDs used in the device.

The electronic driving unit sequentially switched the single-colour LEDs on/off; meanwhile, the simultaneous reading of the voltage from the photodetection unit was carried out. A set of six LEDs was mounted on a separate electronic board. Each LED was coupled via an SMA connector to the fun-outs of the fibre-optic bundle. The results described in [21] showed that the spectrum range allowing the proper classification of samples is the VIS range; therefore, there were selected LEDs that roughly covered that band. The dominant wavelengths and FWHM of each LED are shown in Table 1, while the spectral distributions of all LEDs and their optical powers are shown in Figure 2.

All LEDs were hosted in the same package type, standard TO-18 with a glass lens to allow for easy dismounting and changing when needed.

**Figure 3:**
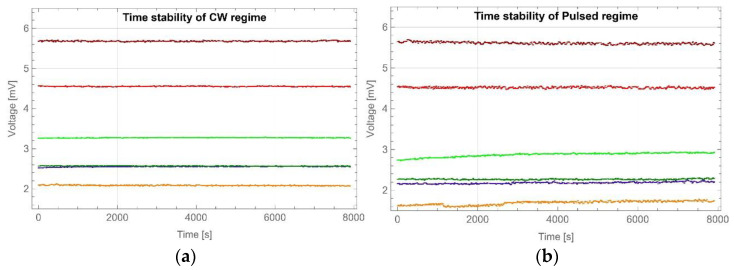
Scheme of signal intensity change in time for: (**a**) continuous (CW) and (**b**) pulsed LED regimes.

The long-term LED performances were tested for continuous and pulsed regimes, and results are shown in Figure 3a,b, respectively. For the continuous regime, the highest optical power fluctuations were obtained for a LED generated with a dominant wavelength of 590 nm—up to 2.4% at the beginning of the tested period. Fluctuations decreased with time and stabilised below 1.5%. The fluctuations of the other LEDs stabilised at or below 1% after a quarter of an hour. During the tests of the pulsed regime, analogous results were obtained. Again, LED-generating at 590 nm showed the highest optical power fluctuations—up to 7.5% in the first operating period, stabilising below 5% after half an hour. The 405 nm LED showed higher power fluctuations than the rest, but did not reach 4.25% during the test. Other LEDs, after a quarter of an hour, stabilised fluctuations at a level below 3%.

**Figure 4:**
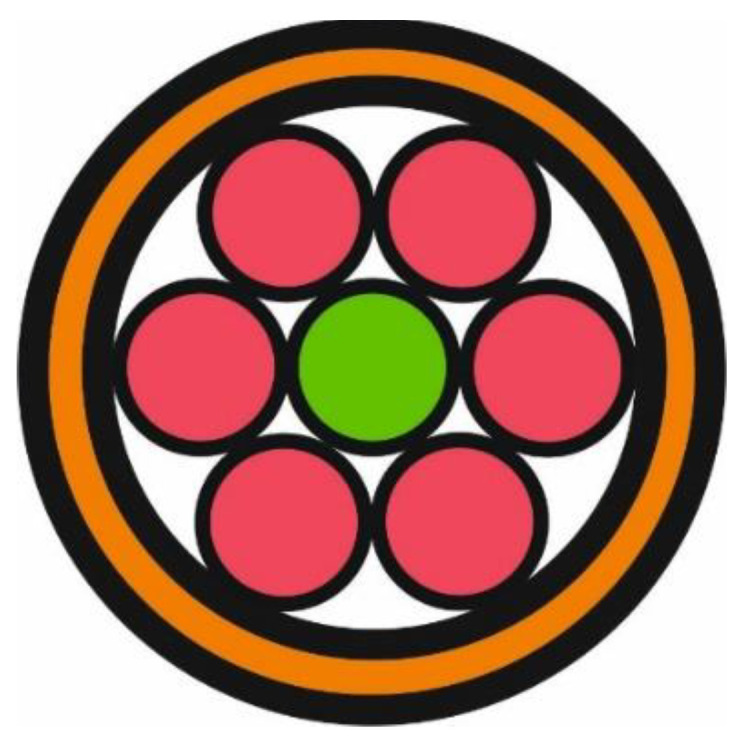
Scheme of a 1-to-7 fun-out fibre-optic bundle in a round-end configuration. The central core of the fan-out (green colour) transfers a back-reflected signal form the measuring head to the photodetector, while other cores (red colour) illuminate the tested sample.

As was mentioned in the previous paragraph, the tested object—a piece of eggshell—was placed on the measuring head and secured only by gravitational forces, as is shown in Figure 1b. The measuring head consisted of a standard 1/2” lens tube which contained an achromatic collimating lens (F950SMA-A, *Thorlabs*) and an output of a 1-to-7 fun-out fibre-optic bundle (BF76LS01, *Thorlabs*). The fan-out had a round-end configuration connected to seven optical fibres with core diameters of 600 μm. The scheme of the fibre-optics bundle is shown in Figure 4. Six externally located fibres (red colour in Figure 4) were connected to different LEDs and illuminated the sample through a collimator. An optical fibre centrally located (green colour in Figure 4) gathered the back-reflected light from the eggshell and transferred it to the photodetector.

**Figure 5:**
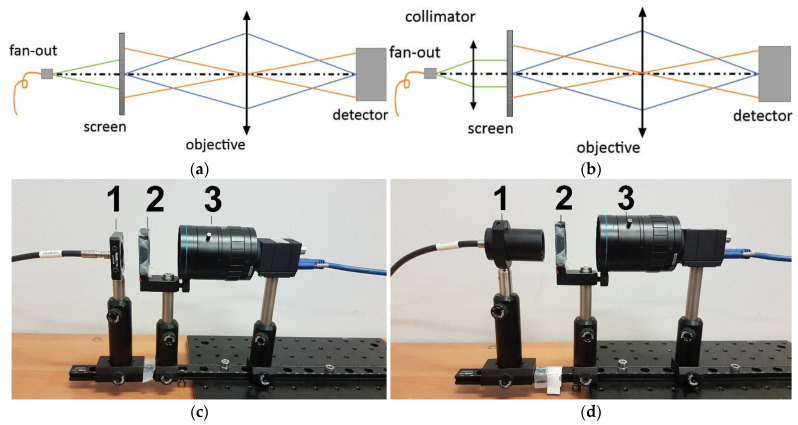
Schemes and images of a laboratory setup build for the performance of the measuring head tests (**a**,**c**) without the collimator and (**b**,**d**) with the collimator.

The critical point of the measurement is the adjustment of the collimating lens and the output of the fan-out. The performance of the measuring head was tested with and without an achromatic collimator. Due to the use of the LEDs generating a very broad spectrum, fibres with a relatively large core diameter and the fibres location in spaces in a round-end configuration, differences in illumination areas for different sources, such as size and location, were expected. The main problem was illuminating the spot evenly on the sample from which the signal was reflected. Therefore, the performance of the measuring head with and without a collimator was tested in a laboratory setup as is presented in Figure 5.

During the calibration procedure, a white ping-pong ball with a rough and clear white surface was used instead of a tested object. This allows for the adjustment of the light intensities in all of the spectral channels accurately. The tested objects (eggshells and a calibration ball) were partially transparent; therefore, the use of an overhead cover/shade was necessary for limiting the influence of external parasitic light on the measurements. The measuring head shaped the beam whilst illuminating the test object and collecting the signal reflected from its surface to the central core of the fibre bundle. An aspherical achromatic collimator, attached to the fibre bundle via the SMA connector, performed the beam-shaping/-gathering roles. It was mounted inside the sleeve that allowed for a smooth object–collimator distance adjustment. The PDA 100A-EC (Thorlabs) with a built-in amplifier served as a detection unit and allowed for the output signal range to be adjusted. The output signal from the photodetector was a voltage signal. The voltage on the photodetector was measured by a digital oscilloscope and was then pre-processed by dedicated software on the PC to be further analysed by a machine-learning algorithm.

**Figure 6:**
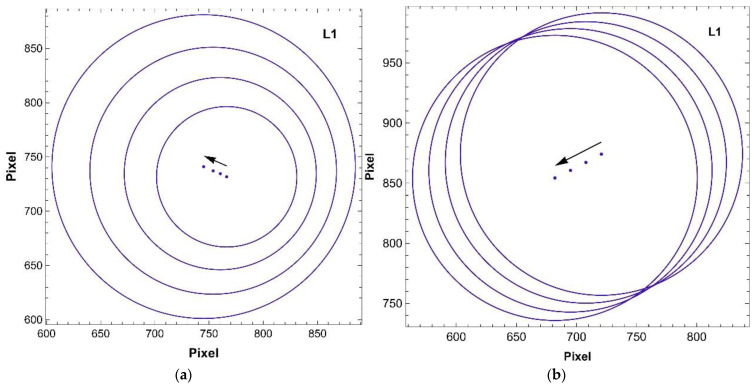
L1 (413 nm) LED illumination spot changes represented by the position of the centre of illumination spot (dots) and the size of the beam diameter at FWHM (circles) tested for four different distances between the fan-out (1) and screen (2) (Figure 5) (**a**) without the collimator and (**b**) with the collimator. Black arrows point out the translation direction of the illumination spots when the distance increased.

For the selected distances between the fan-out/collimator (1) and the screen (2), images of the illumination spot were captured. The distances between the screen (2), the objective (3) and the objective parameters were fixed during the measurement. In both configurations, all LEDs spots were recorded. Each captured image was analysed using standard image-processing algorithms. The following parameters were calculated: the centre-of-illumination spot and the beam diameter at half maximum width (FWHM). The measurement was carried out for an initial distance between the fan-out/collimator (1) and the screen (2) of 22 mm and repeated three times with the step of 2 mm. In the Figure 6, the exemplary results for L1 (413 nm) are shown. When a bare fan-out (Figure 6a) is used, the diameter of the illuminating spot increases with distance due to a high numerical aperture of the fibre NA = 0.39. Moreover, the translation of the centre of the illumination spot is caused by imperfections of the mechanical elements of the laboratory setup (Figure 6a). Due to the fact that the portable optical fibre system proposed in the paper worked in reflection mode, the high convergence of the illumination beam caused a rapid decrease in the measured signal intensity. Therefore, the use of a collimator at the output of the fan-out was proposed. With this configuration, the spot diameter was constant over the measurement distance (Figure 6b). The centre-of-illumination spot shown changed its position due to mechanical imperfections of the measurement setup.

**Figure 7:**
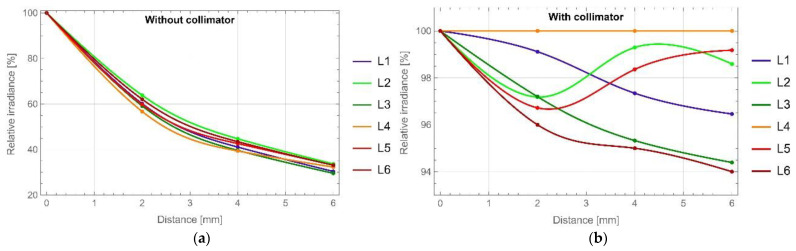
Relative LED irradiance differences are dependent on the distance measuring head–object; measuring head (**a**) without and (**b**) with the collimator.

The measuring head performance was further explored. The overlapping of the illuminated and tested spots was calculated from the experimental data as a function of distance: measuring head–object. As can be expected, the overlapping of the areas increased with the increase in distance. From a starting position of 22 mm, which was the minimum distance set, the overlapping was 69%, and it increased to 85% at a distance increase of 2 mm to reach the full coverage at a distance increase of 4 mm. Due to the numerical aperture of the fibres in the fibre bundle NA = 0.39, the illumination beams spread quite rapidly without using a collimator, which resulted in a rapid decrease in the average irradiation of the tested spot. In the case of a measuring head without a collimator, increasing the distance by 2 mm resulted in a signal drop of 33–47%, while increasing the distance by 4 mm resulted in a signal drop of 55–60%, and the largest distance of 6 mm caused a signal drop of 66–70% depending on the LED. All irradiance differences were calculated as the relative change of signal at the initial position for each LED. Figure 7a,b show the changes of the signals in the tested spot due to the distance change between the measuring head and the object for a measuring head without a collimator and equipped and with a collimator, respectively. There was no visible trend for the measuring head with a collimator, only fluctuations not exceeding 6% for L6 LED of the initial signal, which were linked with the power fluctuations observed during long-term tests.

The use of a collimator allowed for the illumination of the measurement spot irrespective of the distance of the collimator object. In the final configuration of the measuring head, the eggshell was at a distance of 22–28 mm from the collimator. In this case, the illumination spot had a constant diameter of around 1 mm.

## 3. Signal-Processing and Experimental Results

The eggshell/ping-pong ball was placed on the measuring head, and the LEDs were turned on/off in sequence (Figure 1). Each LED light was introduced to the sample through the fibre and collimator. It reflected from the object’s surface and was gathered back by the collimator, and through the central fibre in the bundle was guided to the photodetector. As a result of a single measurement, the sequence-of-voltage peaks were obtained. Each peak corresponded to the back-reflected signal for an individual single-colour LED. The device was calibrated before measurements to prevent the over-saturation of the PDA and allowed for the comparison of the detected signals. The ping-pong white ball was used as a calibrating object.

The constant detector gain in a continuous operation mode was used through the measurement. The detector recorded the values of the beam reflected from the test object and the background signal while all of the LEDs were off. Considering the latter, the specific signal flow was proposed.

**Figure 8:**
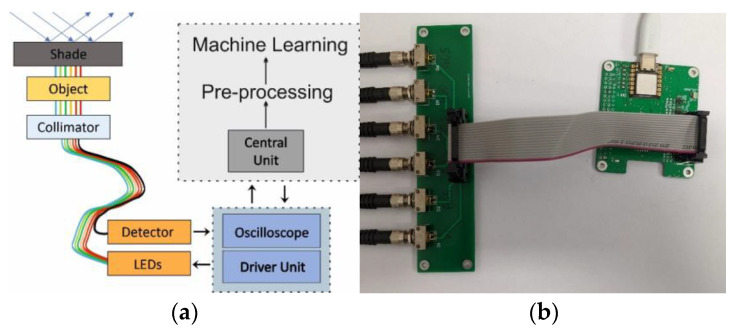
Scheme of the reflectometer system with indicated optical and electrical signals flows (**a**) and an image of driver unit with integrated LEDs and fan-out SMA input connectors (**b**).

The communication between the reflectometer components depended on to their characteristics and how they were implemented in the circuit, as is presented in Figure 8. The control algorithm on the driver unit initiated the diodes. The selected LED on the LEDs module was switched on and the light beam was transmitted by a corresponding fan-out fibre and. through the collimator. illuminated the test object. The collimator, as was already mentioned, modulated the geometric shape of the beam and allowed for the maximising of the measurement signal. The partially reflected and scattered beam entered the middle fibre of the bundle and was directed to the detector. The irradiance of the collected signal was registered by the photodetector and recorded by an oscilloscope. The oscilloscope recorded a series of consecutive measurements of different LEDs and saved them to a file. This data served as the basis for further numerical analysis.

**Figure 9:**
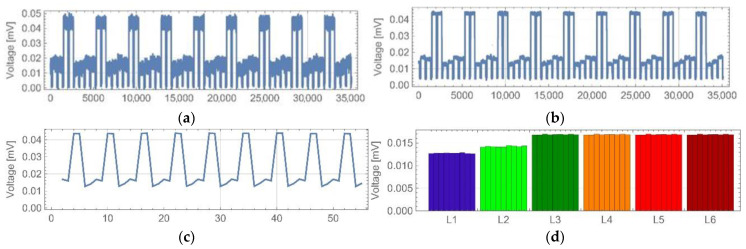
Exemplary output data from the series of measurements: (**a**) registered waveform, (**b**) waveform with determined borders, (**c**) averaged pulses and (**d**) series of pulses assigned to corresponding LEDs.

The implementation of the machine-learning algorithms required proper data recording. The way the oscilloscope detected the waveforms implicated the pre-processing signal path. The detected signal was recorded continuously. The analysis was considered in two parts: the averaging of the recorded waveforms and the numerical pre-processing of the results. The main stages of the analysis are shown in Figure 9. The averaging algorithm started with the proper loading of the data. Previously saved data files were loaded (Figure 9a) and analysed by the algorithm as one category of objects. Next, the waveform averaging was conducted in the following steps: the determining of the border points of the waveforms pulses, the averaging of the pulses values, the assigning of the pulses to the corresponding LEDs and user verification. Due to the noise, the waveform needed to be averaged before the borders between the pulses and breaks could be determined (Figure 9b). The border-points determinations were based on the accepted signal-break sequence. This algorithm ensured that each signal was followed by a break of a known time value, corresponding to the background noise of the signal. This method was based on searching for rising and falling slopes of the signal. When the limits of the pulses in the waveform were known, the pulse-values-averaging process was applied (Figure 9c). Using the known pulse-limit points, the averaged values of the registered signals were obtained by calculating the geometric mean of the points between successive limit points. The averaged data were assigned to the corresponding LED sources (Figure 9d). The final step in the described algorithm was data displaying for verification by the user, because the loaded data may have contained a writing error or a coarse error.

**Figure 10:**
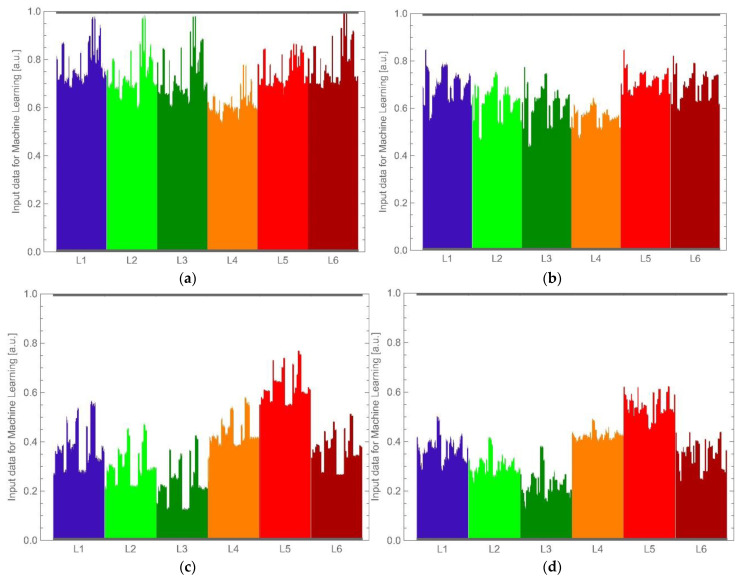
Exemplary final data graph for the samples of the categories (**a**) “healthy white”, (**b**) “infected white”, (**c**) “healthy brown” and (**d**) “infected brown”.

The numerical pre-processing of the results was a practical implementation of the adopted measurement methodology. It was carried out in steps: the measurement of the reference object, which used the entire waveform-averaging patch described above, and the measurement of the biological object, the normalization of the measurement results of the biological objects by the measurement result of the reference object within the range of 0–1. This last step allowed for the elimination of the influence of different optical powers of the illuminating LEDs. Examples of the final input data for machine-learning as graphs are shown below in Figure 10.

## 4. Data Analysis and Discussion

The full analysis of the collected data was performed in the Python environment—v.3.7.4. Due to the fact that the empirical identification and classification of samples—healthy and MS-infected hens—was not possible, the authors decided to use machine-learning for this purpose. The support vector machine (SVM) algorithm was used. It is one of the most popular solutions within the supervised learning area and was developed by Cortes and Vapnik [24]. SVM is a classifier which aims to find a suitable hyperplane that separates a multi-space dataset into two classes. It determines the location of the hyperplane based on the extreme points of the dataset. These points are called support vectors. The separation of the classes is carried out with a maximum margin. The SVM is used for linearly and non-linearly separated data. In the first case, a single straight separates the dataset into two classes; for non-linearly separated data, the solution is much more complex. In such a case, the dimensions of the hyperplane depend on the number of features included in the dataset. Different kernel functions are used along with SVM, such as polynomial or hyperbolic tangents; the radial basis function (RBF) kernel is the best choice for practical applications [25,26,27]. The discussed case used the RBF kernel to analyse the measurement data.

To assess the prediction quality of the machine-learning algorithm, the following metrics were employed: precision, recall, and F-score. All of them were based on the following values: TP—true positives, TN—true negatives, FP—false positives and FN—false negatives [26].

- Precision indicates how well the algorithm handles the correct classification of instances by relating the correct detections to all assigned to a given group. Precision is calculated as follows: Precision = TP/(TP + FP).
- Recall is calculated similarly to the above, but relates to all of the elements that should have been assigned to the group. Recall is calculated as follows: Recall = TP/(TP + FN).
- The F-score is calculated based on precision and recall. It is their harmonic mean. The F-score is calculated according to the following equation:
  F-score = 2(Precision∙Recall)/(Precision + Recall). With this metric, it is easy to detect that one of the coefficients is too low, due to the fact that a low value of one of the coefficients strongly affects its value.

The data were randomly divided in a ratio of 7:3 into the parts required for the learning and model verification. In order to avoid random erroneous results, the learning and verification calculations were repeated a hundred times, and then the mean values of the resulting parameters were determined. During the calculations, the data was randomly partitioned each time. The data were analysed using three different paths to see how normalising the data at a particular stage affected the final result. In the first approach, they were normalised even before they were separated (“common data normalisation”). In the second approach, the normalisation of the learning and test sets was carried out independently after they were separated (“independent data normalisation”). The third solution was similar to the second, except that the normalisation parameters of the learning set were used in the normalisation of the test set (“dependent data normalisation”). This case reflects the situation when the data collected during the measurement go beyond the range of the data that were used during the learning process.

The results of the eggshells origin classifications quality for white and brown eggs obtained using the described portable multispectral fibre-optic reflectometer and the SVM algorithm are shown in Table 2.

The F-score values for case one (common data normalisation) and case three (dependent data normalisation) are practically the same. Such a case may be interpreted as an asset. It shows that the system based on data falling within a specific range has the right to work correctly even if the system gain changes slightly. When averaging the F-score values for eggshells from healthy and infected individuals within white and brown eggs, the values are white—0.87 and brown—0.96.

## 5. Conclusions

In the paper, the first experimental results of using the multispectral portable fibre-optic reflectometer for chicken eggshell origin classifications in case of MS infections were reported. The application of the fibre bundle allowed for the making of this portable sensor and for the flexibility of the access to the measuring object. The use of LEDs with optimal spectral characteristics reduced the weight and size of the system. Moreover, LEDs made it possible to simplify the optical part of the sensor device and to reduce the complexity of the data processing/machine learning. The presented sensor, together with the proposed signal pre-processing path and machine-learning algorithm, proved to be a powerful tool that can be used for the proper classification of the origin of chicken eggshells. The measurement time of the proposed reflectometer compared with the culture methods based on PPLO broth was significantly shorter, with a single measurement cycle taking less than 1 s; however, up to now it was not a subject of optimalization. Additionally, the obtained results of the F-score factor are higher than those obtained in previous studies [21].

The operating mechanism of the sensor is simple enough to be applied in the field after some minor alternations and further industrial testing.

## Figures and Tables

**Table 1:** Dominant wavelengths and FWHMs of the LEDs used in the portable multispectral fibre-optic reflectometer.

LED #	Dominant Wavelength λ [nm]	FWHM [nm]
L1	413	18
L2	504	34
L3	533	34
L4	604	14
L5	660	20
L6	699	24

**Table 2:** Quality of the origin classifications of white and brown eggs obtained using the described portable multispectral fibre-optic reflectometer and the SVM algorithm. I.—eggshells obtained from infected hens, H.—eggshells obtained from healthy hens.

Common Data Normalisation				
Eggshell Colour.	Origin	Precision	Recall	F-Score
white	I	0.81	0.91	0.86
	H	0.92	0.83	0.87
brown	I	0.97	0.95	0.96
	H	0.95	0.97	0.96
**Independent Data Normalisation**				
white	I	0.74	0.79	0.76
	H	0.83	0.76	0.79
brown	I	0.95	0.92	0.93
	H	0.92	0.95	0.93
**Dependent Data Normalisation**				
white	I	0.82	0.92	0.86
	H	0.92	0.83	0.87
brown	I	0.96	0.95	0.96
	H	0.95	0.97	0.96

## Data Availability

The data presented in this study are available on request from the corresponding author. The data are not publicly available due to privacy issues.
